# Development of a novel atrophic non-union model in rabbits: A preliminary study

**DOI:** 10.1016/j.amsu.2021.102558

**Published:** 2021-07-14

**Authors:** Khan Sharun, Abhijit M. Pawde, Amitha Banu S, K.M. Manjusha, E. Kalaiselvan, Rohit Kumar, Prakash Kinjavdekar, Amarpal

**Affiliations:** Division of Surgery, ICAR-Indian Veterinary Research Institute, Izatnagar, Bareilly, Uttar Pradesh, India

**Keywords:** New Zealand White rabbits, Rabbit, Radius, Non-union model, Atrophic non-union model

## Abstract

**Background and aim:**

The currently available atrophic non-union models rely on wide segmental excision of bone diaphysis to impede the process of healing but lack resemblance to the clinical scenario. The present study focused on developing an *in vivo* model of atrophic non-union fracture in rabbit radius that can replicate the clinical scenario.

**Materials and methods:**

The atrophic non-union fracture model was developed by creating a 10 mm segmental bone defect in the radial diaphysis of five adult New Zealand White rabbits. The periosteum (2 mm) of the cut bone ends was cauterized using electrocautery to induce atrophy. Atrophic non-union was confirmed using radiographic and histologic evaluations on 30th postoperative day.

**Results:**

The radiographic signs of healing were completely absent in all the rabbits on 30th postoperative day, indicating inert bone ends. Histological findings further confirmed the presence of inert bone ends, indicating the development of atrophic non-union.

**Conclusion:**

The combination of the segmental bone defect, electrocautery induced thermal damage of bone end periosteum, and delayed treatment can induce the development of atrophic non-union fracture model in rabbits that can replicate the clinical scenario.

## Highlights

• *In vivo* model of atrophic non-union fracture in rabbit radius was developed that can replicate the clinical scenario.
• Radiographic and histological findings confirmed the presence of inert bone ends.
• Combination of segmental bone defect, electrocautery induced thermal damage, and delayed treatment can induce atrophic non-union fracture.

## 1 Introduction

Fracture healing generally proceeds in an orderly fashion under an appropriate biological and mechanical environment, and consequently, most fractures heal well. However, complications like osteomyelitis, implant loosening, and fracture instability may result in a non-union fracture [1]. Approximately 5–10% of fractures fail to heal in human patients, resulting in fracture non-union [2]. Fracture non-union is a pathological condition in which the ends of the fractured bone cannot unite without any surgical or non-surgical intervention [3]. Some of the local factors that have a significant role in the development of non-union include infection, biomechanical instability, iatrogenic factors, decreased vascularity, poor bone fragment contact, and high magnitude of injury [4]. The treatment of fractures that develop non-union may require numerous interventions with associated morbidity and financial costs [5].

Non-union of the radius-ulna fractures commonly results due to instability and macro-movement at the fracture site resulting mainly from stabilization with external coaptation, use of intramedullary pins with inadequate rotational and axial stability, use of external skeletal fixator with improper frame and pin size, loose cerclage wire that has migrated into the fracture site and plates and screws that are inadequately sized for the patient [6]. According to Weber and Cech (1976), fracture non-unions are classified into two broad groups, hypertrophic and atrophic non-unions, based on the radiographic appearance, correlated with its etiology [7]. In hypertrophic non-union, there will be normal callus tissue, but bony bridging will be absent. While in the case of atrophic non-unions, there will be a complete absence of relevant callus tissue. Furthermore, hypertrophic non-union occurs when there is a lack of fracture stability, while atrophic non-union can occur due to several causes [8].

The mechanism responsible for the development of atrophic non-union is not well understood. The lack of suitable animal models significantly limits studies investigating the formation of atrophic non-union and their management. Atrophic non-union models have been created by producing either extensive segmental bone excision or by implanting inert materials within bone defects to impede the normal healing processes [9]. The majority of the non-union models studied for evaluating bone healing used the principle of the critical-sized defect (CSD). It involved creating a large segmental defect beyond the regenerative capacity of the bone [10]. However, such non-union models seemed to be well vascularized at the defect site thereby cannot be used to mimic clinical scenario. Thus, instead of implanting the biomaterials into the fresh-cut segmental defect, delaying the implantation procedure can simulate the clinical scenario where the bone edges become inert and lack vascularization.

Therefore, the present study was conducted to develop an *in vivo* model of atrophic non-union fracture in the radius bone of New Zealand White rabbits that can replicate the clinical scenario.

## 2 Materials and methods

### 2.1 Ethical approval

All experimental protocols used in the study were according to the international guidelines and were approved by the Institute Animal Ethics Committee, ICAR-Indian Veterinary Research Institute.

### 2.2 Experimental animals

Five clinically healthy adult New Zealand White rabbits (n = 5, three male and two female) of seven to eight months of age were used in the study. The rabbits had an average body weight of 2.04 ± 0.09 kg. They were housed in individual steel cages, fed a standard diet (18% crude protein and 2700 kcal digestible energy), and provided *ad libitum* access to drinking water. The rabbits were also given an acclimatization period of 15 days before initiation of the study. The experimental animals were provided with humane care, and procedures were performed according to National Institute of Health (NIH) guidelines for the care and use of laboratory animals (NIH Publications, eighth edition, 2011).

### 2.3 Preparation of atrophic non-union model in rabbit

**Fig. 1:**
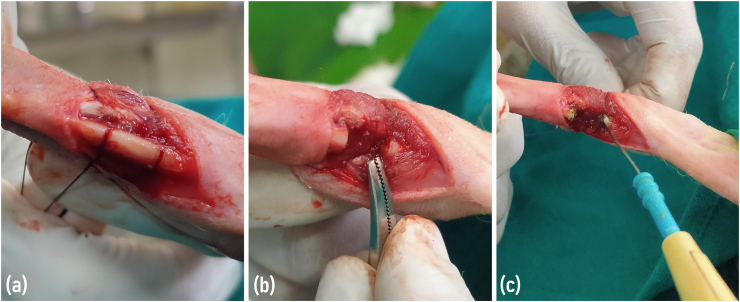
**(A)** Segmental bone defect of 10 mm length was created in the central diaphysis of the radius bone. **(b)** The edges of the bone defect after removing the cut fragment. **(c)** Cauterization of the periosteum using electrocautery circumferentially for a distance of 2 mm on the remaining proximal and distal bone ends.

The rabbits were anesthetized by intramuscular injection of xylazine hydrochloride (Xylaxin, Indian Immunologicals Ltd., India) at a dose rate of 6 mg/kg body weight followed by ketamine hydrochloride (Aneket, Neon Laboratories Ltd., India) at the dose rate of 60 mg/kg body weight in the thigh muscles [11]. The entire forelimb of the rabbit was shaved and painted with 10% povidone-iodine (Betadine, Win-Medicare Pvt. Ltd., India). The rabbits were restrained in lateral recumbency on a surgical table. A three cm long skin incision was made on the medial aspect of the limb in between the elbow and carpal joint. The muscle layers were separated using fine artery forceps to visualize the radius bone. A segmental bone defect of 10 mm length was created in the central diaphysis of the radius bone using an orthopedic saw (Fig. 1a and b). The periosteum was cauterized using electrocautery (Digital 400, Larsen & Toubro Ltd., India) circumferentially for a distance of 2 mm on each end of the remaining proximal and distal bone segments (Fig. 1c). Care was taken to protect the muscle and other surrounding soft tissues around the fracture site.

The muscle layers and skin were closed in a routine manner. The defect produced was left untreated for a period of 30 days. Post-operatively, the rabbits were treated with antibiotic enrofloxacin (Quinintas, Intas Pharmaceuticals Ltd., India) at the dose of 5 mg/kg body weight IM q24 h for five days and anti-inflammatory agent meloxicam (Melonex, Intas Pharmaceuticals Ltd., India) at the dose of 0.5 mg/kg body weight IM q24 h for three days. Immediately after the surgery, an external bamboo splint was applied to the test limb to provide good immobilization and prevent excessive weight-bearing. The confirmation of non-union was done based on radiographic findings after 30 days.

### 2.4 Radiographic evaluation of the bone defect healing

Atrophic non-union was confirmed based on the findings of the radiographic examination. Postoperative radiographs of the defect site (mediolateral view) were made immediately after surgery and subsequently on the 30th postoperative days using standard exposure factors (14 mAs, 50 kVp, and 85 cm FFD). The radiographs were evaluated for the degree of new bone formation, the extent of callus formed, radiographic density, reduction in the defect size, and the signs of remodeling.

### 2.5 Histological evaluation

For histological evaluation, one of the rabbits was sacrificed on the 30th postoperative day. The test bone was collected, and a three cm long piece of the radius that included the defect site at the middle and normal bones on both sides was cut using a hack saw. The bone specimens were washed and then fixed using 10% formalin for a period of 2–3 days. The specimens were then decalcified by using Goodling and Stewart's fluid [12]. The solution was stirred daily and changed once every three days. The bone samples were checked regularly for the signs of complete decalcification by observing different parameters like flexibility, transparency, and pin penetrability. The decalcified bone was further processed by paraffin embedding technique to obtain 4-μm thick paraffin sections (longitudinal sections). The bone sections were stained using Haematoxylin and Eosin (H & E) to evaluate the healing status of the bone [13].

## 3 Results

### 3.1 Clinical signs

None of the rabbits exhibited abnormal behavior/activity during the postoperative period except that they did not bear weight on the affected limb (right forelimb). Application of splints also caused inconvenience and improper weight-bearing on the affected limb. However, regular weight-bearing was observed after 10–14 days when the external bamboo splint was removed. The surgical wound in all the rabbits healed without any complications.

### 3.2 Radiographic observations

**Fig. 2:**
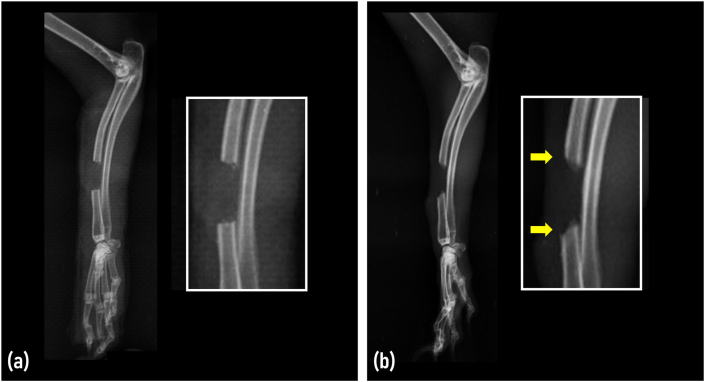
Mediolateral radiographs of the rabbit showing the status of bone defect at **(a)** day 0 and **(b)** day 30. Radiographic features on day 30 include reduced bone density and bone end shortening (arrows).

Radiographic examination was performed on the day of surgery and on day 30 to evaluate the healing process and progression of atrophic changes in the bone edges. The radiolucent area at the mid-diaphysis was visible on day 0, representing the 10 mm segmental defect. The rabbit radius measured a mean diameter of 3.02 ± 0.13 mm (range 2.8 mm–3.1 mm). In all the experimental rabbits, signs of new bone formation were completely absent, indicating inert bone ends. Furthermore, radiographic features such as end shortening and reduced bone density were also evident in all rabbits at the bony ends on day 30, indicative of atrophic non-union fracture. Fig. 2 illustrates the comparative analysis of the day 0 and day 30 radiographs of the right radius (mediolateral view).

### 3.3 Histological observations

**Fig. 3:**
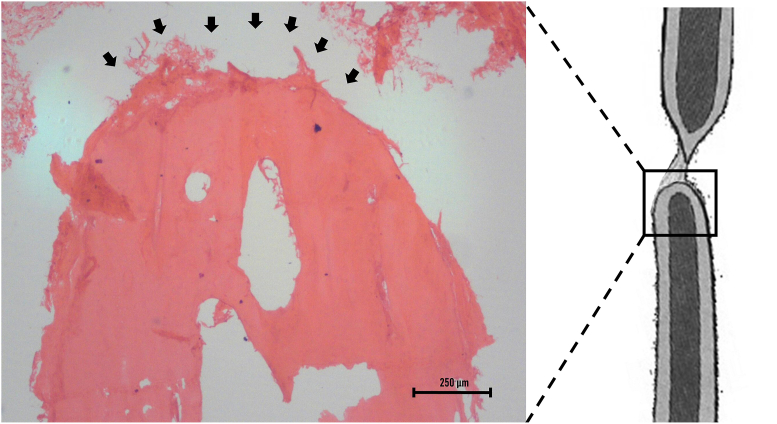
Photomicrograph showing the histological picture of the bony end (arrows) showing atrophic changes in the longitudinal section of the radius on day 30 (H & E stain; x4).

The histological picture of the bony ends indicated atrophic changes in the longitudinal section of radius on day 30 (Fig. 3). The protocol used in this study successfully prevented the end-to-end union of the radius bone. Instead, the gap defect was found to be filled with fibrous tissue. The absence of osteocytes in the diaphysis immediately adjacent to the bony ends indicated inertness. Therefore, the histopathological findings confirmed the formation of atrophic non-union fractures.

## 4 Discussion

In hypertrophic non-union, callus formation is abundant due to its good biological activity. The fracture cannot heal properly due to a lack of mechanical stability [14]. Compared to hypertrophic non-union, atrophic non-union is a pathological condition that occurs from biological impairment of healing characterized by the absence of the callus at the fracture ends, making it rounded and sclerotic [15]. Treatment of non-union is considered a difficult task and is associated with a high financial impact [8]. Several novel therapeutic strategies like extracorporeal shock wave therapies are available that mainly target hypertrophic non-unions. Such therapies are less effective in managing atrophic non-unions [16].

The disease model of atrophic non-union should accurately reflect the abnormal physiological process of fracture healing. Selecting a suitable non-union model should involve specific considerations like animal species, types of fixations, and mode of generation of the atrophic non-union. Small animals are widely used in studies of fracture healing due to several obvious advantages over large animals that include lower maintenance costs, shorter experimental time, and the possibility of using many animals. The process of fracture healing has been studied extensively in small animals such as mice and rats to understand the same process in human beings [17,18]. Atrophic non-unions can be confirmed from the typical histological and radiological features. The characteristic findings include the absence of fracture bridging, the absence of callus formation, an abundance of fibrous tissue at the fracture gap, and the rounding of bone ends [19].

The actual meaning of atrophic non-unions is described as the presence of non-reactive and metabolically inactive bony ends. Research on non-unions is greatly limited by the lack of an ideal animal model. Among different animal models, the rabbit model is considered a perfect choice for studies involving tissue engineering of cartilage, bone, tendon, and skin [20]. It is mainly because of the advantages like easy standardization of experimental conditions, less expensive, and superior bone turnover rate [21]. It also has a well-defined cortical bone Haversian system, and the simulation of remodeling after an injury is more similar to that of other large animals [22]. Over the years, several non-union models have been developed that closely resemble the clinical scenario. Some of which include segmental bone excision [23,24], external skeletal fixation with periosteal stripping [9], bone plating with periosteal stripping [25,26], sealing of the marrow cavity [27], removal of the bone marrow [28], and by placing a spacer in the defects to create a delay in bone union [29]. The successful creation of non-union in the animal model depends largely on the size of the bone defect. The length of CSD in the rabbit radius can also be calculated by formula 2n+1, where n is the diameter of the radius bone in mm [10]. The defect made in the present study was approximately three times greater than the diameter of the radius bone, thereby conforming to the definition of CSD. Even though previous studies have identified that radial osteotomy defects up to 15 mm can be healed with the help of suitable therapeutic interventions [30], we firmly believe that the length of defect used in our study (10 mm) can be further decreased without affecting the atrophic non-union formation. In addition to the osteotomy, the fracture ends have to be de-vascularized to promote the formation of non-union. This can be done by cauterizing the periosteum at the fracture ends [31]. Kokubu et al. (2003) cauterized 2 mm of the periosteum on each side of the fractured femur in an atrophic non-union rat model [32]. Therefore, we have also cauterized 2 mm of the periosteum on the remaining proximal and distal bone ends. Cauterization can simulate clinical atrophic union as it can promote cortical necrosis [33].

In the case of rats, muscle interposition was performed into the fracture site to induce the formation of non-union [34]. Damaging both periosteum and endosteum is another strategy that has been used widely in rats to induce non-union [35,36]. Similarly, extensive resection of periosteum and bone marrow is another method of inducing an atrophic non-union model in rat femur [37]. The non-union model can also be created in mice using a pin-clip fixation technique [19]. However, most of these models did not produce an accurate representation of the atrophic non-union.

The majority of the non-union models used for evaluating bone healing use the principle of CSD and are made by creating a large segmental defect beyond the regenerative capacity of the bone. It is followed by implanting suitable biomaterials into the freshly cut CSD [10]. Those defects that cannot heal completely within the lifetime of the animal without any additional therapeutic interventions are defined as CSD [38]. Radius is the ideal bone in rabbits that can be used for evaluating segmental bone defect due to its several privileges: it is a tubular bone that enables easy creation of defect, radiographical and histopathological evaluations are easy; simplified surgical procedure, no need for additional fixations since ulna provides support and stability [39].

In the present study, atrophic non-union fracture was created in the radius bone by combining segmental bone defect, periosteal damage using electrocautery, and delayed treatment. The fracture site was filled with fibrous tissue, which was evident in the histopathological investigation performed on day 30. This was in accordance with the findings of Onishi et al. (2020) [37]. We have developed an atrophic non-union model in rabbits that mimics the clinical scenario of non-unions. Atrophic non-union was confirmed in our study on day 30 due to the lack of fracture bridging, absence of callus formation, and the presence of abundant fibrous tissue in the bone defect. One of the major advantages of this model is the lack of fracture fixing equipment such as bone plates [25,26], external skeletal fixator [9], K-wire [37], and pin-clip fixator [19] for creating non-union. The segmental bone defect in the radius was already fixed and stabilized due to the adjacent ulna. This allowed full weight-bearing in the rabbits and prevented the displacement of fracture ends. The lack of proper immobilization would have probably led to the development of pseudarthrosis [28].

## 5 Conclusion

The combination of the segmental bone defect, electrocautery-induced thermal damage of bone end periosteum, and delayed treatment can induce atrophic non-union fracture of radius in the rabbit model that can replicate the clinical scenario. However, further studies are required to standardize the length of segmental bone defect and the optimum duration of the delay necessary to induce successful atrophic non-union in rabbit radius.

## Ethical approval

All the methods and experimental protocols used in this study were approved by the Institute Animal Ethics Committee (IAEC) of ICAR-Indian Veterinary Research Institute, Izatnagar, Bareilly, Uttar Pradesh, India.

## Trail registry number

Not applicable (no human study).

## Provenance and peer review

Not commissioned, externally peer-reviewed.

## Research Registration Unique Identifying Number (UIN)

Name of the registry: Not applicable

Unique Identifying number or registration ID: Not applicable

Hyperlink to your specific registration (must be publicly accessible and will be checked): Not applicable

## Author contribution

KS, and AMP were involved in conception and design, data collection, analysis and interpretation, writing the article. ABS, KMM and EK participated in the study and analysis. RK, PK, and Amarpal critically revised the manuscript. All authors certifies that he/she has made a direct and substantial contribution to the work reported in the manuscript and have approved the final version of the manuscript

## Data statement

The authors confirm that the data supporting the findings of this study are available within the article.

## Funding

No substantial funding to be stated.

## Guarantor

**Dr. Abhijit M Pawde**, MVSc., PhD, Division of Surgery, ICAR-Indian Veterinary Research Institute, Izatnagar, Bareilly, Uttar Pradesh, India.

## Declaration of competing interest

All authors declare that there exist no commercial or financial relationships that could, in any way, lead to a potential conflict of interest.
